# YAP activity protects against ventilator-induced lung injury

**DOI:** 10.3389/fphys.2025.1578901

**Published:** 2025-10-03

**Authors:** Huan Liu, Xuepeng Yang, Feng Qi, Xuan Li, Yu Liu, Ge Liu, Xiaojie Lin

**Affiliations:** ^1^ Department of Anesthesiology, Qilu Hospital, Cheeloo College of Medicine, Shandong University, Jinan, China; ^2^ Research Center for Basic Medical Sciences, Qilu Hospital, Shandong University, Jinan, China; ^3^ Department of Ophtalmology, Jinan Second People’s Hospital, Jinan, China; ^4^ Department of Anesthesiology, The Second Hospital, Cheeloo College of Medicine, Shandong University, Jinan, China

**Keywords:** YAP, VILI, regeneration, repair, pulmonary fibrosis

## Abstract

**Introduction:**

Mechanical ventilation (MV) activates inflammatory signaling pathways, leading to ventilator-induced lung injury (VILI), the activation of lung repair processes, persistent inflammatory stimulation and incomplete tissue repair leads to pulmonary fibrosis. The role of Yes-associated protein (YAP) in VILI and related tissue repair mechanisms remains elusive.

**Methods:**

We examined the effects of inhibiting or stimulating YAP activity on VILI, lung repair and fibrosis in a mouse model of MV-induced lung injury. Mice were subjected to either low tidal volume ventilation (LVT) or high tidal volume ventilation (HVT), and HVT was used in subsequent experiments. Additional mice were treated with or without the YAP inhibitor verteporfin (VP) and with or without the YAP stimulator XMU-MP-1 (X) and then subjected to HVT. The severity of lung injury and fibrosis was evaluated via histological analysis; the extent of lung repair was tested by measuring the levels of alveolar epithelial cell (AEC) marker proteins; YAP activity was assessed via Western blotting, immunoprecipitation and immunofluorescence.

**Results:**

MV caused lung injury and fibrosis, decreased the protein expression of AEC markers and β-catenin, increased YAP expression, and the effect of HVT was greater than that of LVT. After inhibition of YAP activity, HVT decreased β-catenin expression, further inhibiting regeneration of AECs and worsening lung injury and fibrosis. In contrast, after stimulation of YAP activity, the reduction in β-catenin was mitigated, the impairment of AEC regeneration was ameliorated, lung injury and fibrosis were alleviated.

**Discussion:**

The results indicate stimulation of YAP activity alleviates VILI by promoting lung repair and inhibiting fibrosis development.

## Introduction

During the polio epidemic in Copenhagen in 1952 (Oshinsky, 2023), mechanical ventilation (MV), which allows patients to achieve sufficient gas exchange and relax the respiratory muscles, reduced the case fatality rate from 80% to 40%. At present, MV is an important means of respiratory support in critically ill and perioperative patients, but this treatment strategy can also lead to barotrauma, volutrauma, atelectrauma and biotrauma, which can aggravate lung diseases or harm healthy lungs (Ou et al., 2024). The pathological changes that result from MV, known as ventilator-induced lung injury (VILI) (Ran et al., 2023; Nieman et al., 2022), often include inflammatory cell infiltration, pulmonary edema and transparent membrane formation (Liu et al., 2019). The mechanism of VILI is complex and involves physical and biological mechanisms. Numerous studies have confirmed that the interaction between mechanical stress and immune pathways aggravates lung injury, that mechanical stress destroys alveolar epithelial cells (AECs) and mediates alveolar barrier dysfunction, and that the activation of inflammatory signaling pathways induces the release of inflammatory mediators and cytokines, which can further aggravate alveolar barrier dysfunction, ultimately causing pulmonary edema and lung injury (Curley et al., 2016; Liu H. et al., 2020). In conclusion, the initial cause of VILI is mechanical injury, and the inflammatory response mediated by biological injury plays ultimately results in more severe damage.

AECs can be divided into type I and type II AECs. Type I AECs are large flat cells that participate in the formation of the gas‒blood barrier and occupy more than 95% of the alveolar surface; Type II AECs, which occupy 2%–5% of the alveolar surface, are cuboidal and secrete surfactants that reduce the alveolar surface tension (Wu and Song, 2020). Type I AECs are more sensitive to pressure than type II AECs, and the repair and reconstruction of type I AECs after injury are the keys to maintaining proper AEC function (Desai et al., 2014). However, damaged type I AECs cannot be replaced by the self-division of these cells. Type II AECs are thought to have stem cell-like properties and thus the potential to generate new type II AECs and transdifferentiate into type I AECs (Barkauskas et al., 2013). Studies have shown that the repair of type I AECs in the later stage of lung injury is completely dependent on the proliferation and transdifferentiation of type II AECs. Therefore, maintaining the differentiation ability of type II AECs during the repair stage of lung injury is particularly important. The regulation of type II AEC transdifferentiation is complex and involves many signaling pathways, and there may be synergistic or antagonistic interactions.

The hippo signaling pathway, which was first identified in *Drosophila*, is a highly conserved protein kinase cascade pathway that plays a significant role in the regulation of cell proliferation and differentiation (Lin et al., 2024). YAP is a vital effector molecule of the hippo signaling pathway. Inhibition of the hippo signaling pathway or defects in core members of this pathway leads to the nuclear translocation of YAP, which combines with transcriptional enhanced associate domain (TEAD) family member transcription factors to induce the transcription of cysteine-rich angiogenic inducer 61 (Cyr61/CCN1) and connective tissue growth factor (CTGF/CCN2) and accelerate cell growth, proliferation and survival (Fu et al., 2022). In addition, YAP may protect against lung injury by inhibiting inflammation in the injury phase and promoting lung repair and inflammation resolution in the repair phase (Liu L. Y. et al., 2020). However, the mechanism by which YAP regulates the proliferation and transdifferentiation of AECs is unknown. Accelerating the proliferation and transdifferentiation of AECs in the early stage of lung injury may be an effective therapeutic strategy for VILI.

Diffuse alveolar injury, excessive extracellular matrix deposition, and excessive proliferation of lung interstitial cells lead to irreversible lung injury, namely, pulmonary fibrosis (Tang et al., 2023), which is the terminal stage of VILI. In the presence of many stimuli, AECs are the first cells to be damaged, and type II AECs rapidly self-renew and transdifferentiate into type I AECs. At the same time, interleukins, chemokines and profibrotic factors are activated and released (Distler et al., 2019). YAP can form a complex with members of the TGF-β/Smad signaling pathway, which promotes the expression of CTGF and accelerates the development of fibrosis. Pulmonary fibrosis involves many signaling pathways through mutual regulation. Early treatment of lung injury can result in the acceleration of type II AEC proliferation and the transdifferentiation of these cells into type I AECs to repair the damaged alveolar membrane and inhibit the process of fibrosis to ameliorate VILI.

This study found MV aggravated lung injury accompanied by increased YAP and decreased β-catenin activity, and HVT was more severe than LVT. Inhibition of YAP activity accelerated lung injury by suppressing AEC regeneration, and stimulation of YAP activity alleviated lung injury and promoted the recovery of lung architecture. Stimulating YAP activity can promote the regeneration of AEC, thus alleviating VILI.

## Materials and methods

### Mice

Wild-type male C57BL/6 mice were supplied by Beijing Vital River Laboratory Animal Technology Co., Ltd., and housed under specific pathogen-free conditions. A total of 98 mice (6–8 weeks, 25–30 g) were used for the studies and given free access to food and water. All the animal experiments were approved by the Animal Ethics Committee of Qilu Hospital of Shandong University (DWLL-2023-024), and the mice were euthanized after completion of the study. The mice were anesthetized with pentobarbital sodium (intraperitoneal (i.p.) injection, 60 mg/kg) (Sigma‒Aldrich, United States). Subsequently, the mice were intubated and connected to an animal ventilator set to the following parameters: for LVT (n = 6): 9 mL/kg, 160 bpm, PEEP of 3 cmH_2_O, duration of 2 h; for HVT (n = 6): 28 mL/kg, 60 bpm, PEEP of 3 cmH_2_O, duration of 2 h. The control (C) group (n = 6) was intubated but did not receive MV. The severity of VILI was assessed via hematoxylin and eosin (H&E) staining. According to the results, HVT was used for the subsequent experiments.

Verteporfin (VP) (MedchemExpress) (New Jersey, United States), a YAP-TEAD inhibitor (Barrette et al., 2022), and XMU-MP-1 (X) (MedchemExpress) (New Jersey, United States), which blocks Mst1/2 kinase activity to activate the downstream effector YAP (Wu et al., 2021), were used. The mice were divided into the following eight groups: the VP group (i.p. injection of 100 mg/kg VP 2 h before sacrifice) (n = 10), the VP + H group (i.p injection of 100 mg/kg VP 2 h before HVT for 2 h) (n = 10), the Dv group (i.p. injection of DMSO (solvent for VP) 2 h before sacrifice) (n = 10), the Dv + H group (i.p. injection of DMSO 2 h before HVT for 2 h) (n = 10), the X group (i.p. injection of 1 mg/kg X 2 h before sacrifice) (n = 10), the X + H group (i.p. injection of 1 mg/kg X 2 h before HVT for 2 h) (n = 10), the Dx group (i.p. injection of DMSO (solvent for X) 2 h before sacrifice) (n = 10), and the Dx + H group (i.p. injection of DMSO 2 h before HVT for 2 h) (n = 10). After the above treatment, the mice underwent cervical dislocation and were euthanized, and lung tissues were obtained.

### H&E staining

Lungs were obtained, fixed with 4% formalin, dehydrated and embedded in paraffin, after which they were subsequently stained with H&E after being sectioned into 5 μm slices. The stained sections were examined under a light microscope at a magnification of ×200.

### Lung injury scores

Lung injury scores were calculated to assess lung injury which were based on pulmonary edema, the thickened alveolar septa, alveolar structure disorder, alveolar hemorrhage, transparent membrane formation and infiltration of inflammatory cells (Liu H. et al., 2020), (Zhou et al., 2000). A scale of 0–4 was used to describe the severity of the lung injury, a higher score indicated more severe lung injury, and at least three visual fields in each H&E slide were assessed under a microscope.

### Masson’s trichrome staining

Slices prepared as described above were sequentially placed in Weigert hematoxylin solution for 5 min and differentiated in hydrochloric acid alcohol after sufficient washing. Then, they were placed in Ponceau dye for 5 min and differentiated with 1% phosphomolybdic acid. The slices were subsequently stained with aniline blue and sealed with neutral balsam. Finally, the sections were observed under a light microscope at a magnification of ×200.

### Western blotting

As previously described (Liu H. et al., 2020), lung tissues were obtained after mice underwent the appropriate treatment, and total protein was extracted from the lungs. Equal amounts of protein were separated by 10% SDS-PAGE and transferred to polyvinylidene fluoride membranes. The membranes were blocked with 5% nonfat milk in TBST at room temperature for 2 h and incubated overnight at 4 °C with primary antibodies against YAP (1:200, sc-376830) (Santa Cruz, United States), β-catenin (1:200, sc-7963) (Santa Cruz, United States), and β-actin (1:200, sc-47778) (Santa Cruz, United States). The membranes were washed, incubated at room temperature for 2 h with secondary antibody, and washed again three times. The immunoreactive bands were visualized with Immobilon Western Chemiluminescent HRP substrate (Millipore, United States), and the band intensities were quantified with ImageJ.

### Immunoprecipitation

Lung tissues were lysed in buffer containing a protease inhibitor cocktail, and the supernatants was obtained. Forty microliters of each supernatant was used as input, and the remaining samples were precleared with isotype control IgG together with 20 μL of protein A/G plus agarose beads for 4 h. Then, the samples were incubated with an anti-β-catenin antibody (Santa Cruz, United States) and Protein A/G PLUS-agarose beads at 4 °C with rotation overnight. The supernatants were discarded after centrifugation, and the precipitates were washed with PBS. The immunoprecipitates were subsequently dissolved in loading buffer for immunoblot analysis.

### Immunofluorescence

Lung tissues were cut into 5 μm thick cryosections, which were fixed with 4% paraformaldehyde for 20 min, permeabilized with 0.5% Triton X-100 for 5 min, blocked with 5% bovine serum albumin (BSA) for 30 min, and incubated with anti-YAP (1:100, sc-376830) (Santa Cruz, United States), anti-β-catenin (1:200, sc-7963) (Santa Cruz, United States), anti-CTGF (1:100, sc-365970) (Santa Cruz, United States), anti-AQP5 (1:100, sc-514022) (Santa Cruz, United States) and anti-SP-C (1:100, ab90716) (Abcam, United Kingdom) antibodies diluted in 5% BSA at 4 °C overnight. The samples were subsequently washed and incubated with IFKine Green AffiniPure Donkey Anti-Mouse (1:200, A24211) (AmyJet Scientific, China) or IFKine Red AffiniPure Donkey Anti-Rabbit (1:200, A24421) (AmyJet Scientific, China) diluted in 5% BSA for 1 h. The cryosections were washed, and the nuclei were counterstained with 4′,6-diamidino-2-phenyl indole dihydrochloride (DAPI) for 5 min. Then, the sections were thoroughly washed again and mounted with antifade mounting medium. The slides were observed under a fluorescence microscope (ni-u, Nikon, Japan).

### Statistical analysis

The data are expressed as the means ± standard deviations, and GraphPad Prism 8.0 was used for statistical analysis. Comparisons between multiple groups were made via one-way ANOVA. Post hoc analyses were performed via Bonferroni’s *post hoc* test. Comparisons between two groups were made via Student’s t-test. *P* < 0.05 was considered to indicate a statistically significant difference.

## Results

**FIGURE 1:**
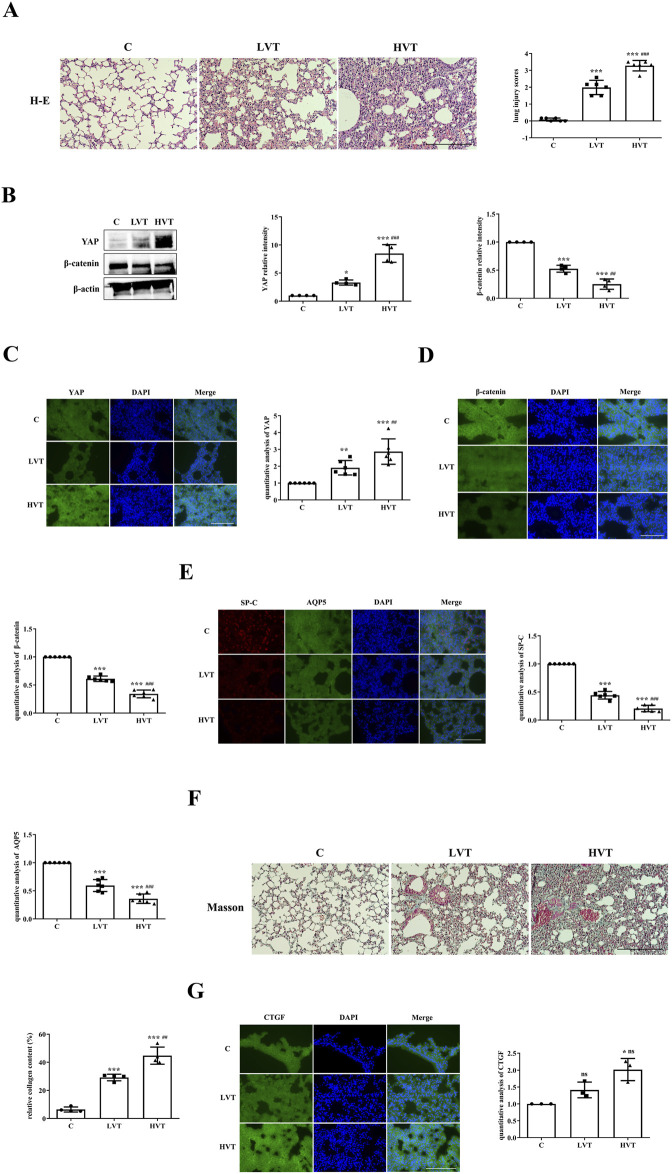
MV caused VILI accompanied by increased YAP activity. **(A)** Histological examination of VILI in mice subjected to LVT or HVT; the degree of lung injury caused by HVT was greater than that caused by LVT. Scale bar = 100 μm; magnification = ×200. The increase of lung injury scores in the C (0.08333 ± 0.09129), LVT (1.986 ± 0.4230), HVT (3.278 ± 0.3103) group indicated a gradual increase in the severity of lung injury, ^***^ *p* < 0.001 versus the C group; ^###^ *p* < 0.001 versus the LVT group. **(B)** The total protein expression of YAP and β-catenin in the lungs was measured via Western blotting. The data are expressed as the means ± SDs, C group (1.000 ± 0.0), LVT group (3.317 ± 0.4566) and HVT group (8.480 ± 1.577), and statistical analysis was performed. ^*^ *p* < 0.05, ^***^ *p* < 0.001 versus the C group; ^###^ *p* < 0.001, ^##^ *p* < 0.01 versus the LVT group. **(C–E)** The expression of YAP (green), β-catenin (green), SP-C (red) and AQP5 (green) was measured via immunofluorescence. Scale bar = 50 μm. The data are expressed as the means ± SDs, the expression of YAP in C group (1.000 ± 0.0), LVT group (1.911 ± 0.4309), HVT group (2.871 ± 0.7525); the expression of β-catenin in C group (1.000 ± 0.0), LVT group (0.6106 ± 0.04893), HVT group (0.3415 ± 0.06941); the expression of SP-C in C group (1.000 ± 0.0), LVT group (0.4419 ± 0.06777), HVT group (0.2062 ± 0.05706); the expression of AQP5 in C group (1.000 ± 0.0), LVT group (0.5947 ± 0.1063), HVT group (0.3594 ± 0.08355). ^**^ *p* < 0.01, ^***^ *p* < 0.001 versus the C group; ^##^ *p* < 0.01, ^###^ *p* < 0.001versus the LVT group. **(F)** Pulmonary fibrosis caused by LVT or HVT was observed by Masson’s trichrome staining; collagen fibers (blue), muscle fibers and erythrocytes (red) are shown. Scale bar = 100 μm; magnification = ×200. C group (6.404 ± 1.765), LVT group (29.17 ± 2.354), HVT group (44.73 ± 6.116). The relative collagen content was quantitatively analyzed, ^***^ *p* < 0.001 versus the C group; ^##^ *p* < 0.01 versus the LVT group. **(G)** The expression of CTGF (green) in C group (1.000 ± 0.0), LVT group (1.414 ± 0.2324) and HVT group (2.016 ± 0.3255) was measured via immunofluorescence. Scale bar = 50 = μm. The difference between HVT and C group was statistically significant, ^*^ *p* < 0.05.

As described in the Methods sections, the severity of VILI was assessed via H&E staining, slight pulmonary architecture destruction, inflammatory cell infiltration, alveolar hemorrhage and alveolar septum thickening were observed in the LVT group compared with the C group, and the severity of damage was greater in the HVT group than in the LVT group, lung injury scores also verified this conclusion (Figure 1A). Thus, HVT was selected for the subsequent experiments. The protein expression of YAP and β-catenin was determined by Western blotting. The expression of YAP was increased by LVT and further increased by HVT, whereas the expression of β-catenin showed the opposite trend (Figure 1B). To confirm the above conclusions, we examined the localization of YAP and β-catenin by immunofluorescence and found that the trend of change was the same as that reported above: specifically, LVT increased YAP levels and decreased β-catenin levels in the cytoplasm, and HVT had a more pronounced effect (Figures 1C,D). MV also altered the expression of the type I and type II AEC markers AQP5 and SP-C. LVT reduced the expression of AQP5 and SP-C, and HVT accelerated the reduction in the expression of these proteins; moreover, MV decreased the expression SP-C to a greater extent than the expression of AQP5 (Figure 1E). Masson’s trichrome staining was used to evaluate the degree of pulmonary fibrosis. Following Masson’s trichrome staining, collagen fibers were stained blue, and muscle fibers and erythrocytes were stained red. HVT caused diffuse perialveolar, peribronchial and interstitial fibrosis, whereas the extent to which LVT caused fibrosis was limited (Figure 1F). As predicted, HVT increased CTGF expression in the cytosol, whereas the effect of LVT was not statistically significant (Figure 1G).

**FIGURE 2:**
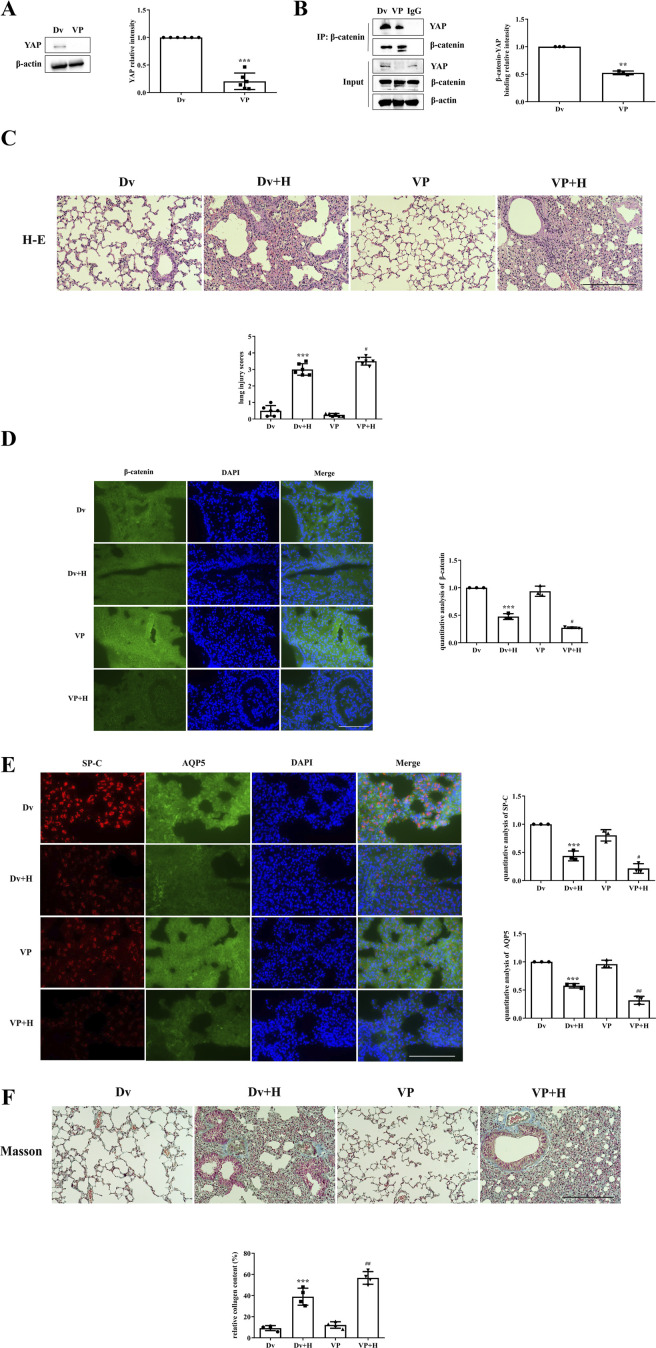
The inhibition of YAP activity worsened VILI. **(A,B)** Mice were intraperitoneally injected with VP, and YAP activity was measured by Western blotting, Dv group (1.000 ± 0.0000), VP group (0.2045 ± 0.1498). Statistical analysis was performed. ^***^ *p* < 0.001 versus the Dv group. The interaction of YAP with β-catenin was also decreased according to immunoprecipitation, Dv group (1.000 ± 0.0000), VP group (0.5247 ± 0.03298). ^**^ *p* < 0.01 versus the Dv group. **(C)** H&E and lung injury scores were used to evaluate the severity of VILI, respectively, in mice subjected to HVT and pretreated with or without VP. Scale bar = 100 μm; magnification = ×200. Dv group (0.5000 ± 0.3162), Dv + H group (3.000 ± 0.3496), VP group (0.2500 ± 0.09129), VP + H group (3.500 ± 0.2357). ^***^ *p* < 0.001 versus the Dv group, ^#^ *p* < 0.05 versus the Dv + H group. **(D,E)** The localization of β-catenin (green), SP-C (red) and AQP5 (green) was observed by immunofluorescence, and the nuclei were stained blue. Scale bar = 50 μm. The data are expressed as the means ± SDs, the expression of β-catenin in Dv group (1.000 ± 0.0), Dv + H group (0.4766 ± 0.05310), VP group (0.9371 ± 0.09235), VP + H group (0.2735 ± 0.01280); the expression of SP-C in Dv group (1.000 ± 0.0), Dv + H group (0.4383 ± 0.08742), VP group (0.8031 ± 0.1009), VP + H group (0.2157 ± 0.08496); the expression of AQP5 in Dv group (1.000 ± 0.0), Dv + H group (0.5767 ± 0.03948), VP group (0.9612 ± 0.06829), VP + H group (0.3183 ± 0.07141). ^***^ *p* < 0.001 versus the Dv group, ^#^ *p* < 0.05, ^##^ *p* < 0.01 versus the Dv + H group. **(F)** Masson’s trichrome staining was used to assess pulmonary fibrosis; collagen fibers (blue), muscle fibers and erythrocytes (red) are shown. Scale bar = 100 μm; magnification = ×200. Dv group (9.173 ± 2.366), Dv + H group (38.92 ± 8.038), VP group (12.14 ± 3.066), VP + H group (56.67 ± 5.968). The relative collagen content was quantitatively analyzed, ^***^ *p* < 0.001 versus the Dv group, ^##^ *p* < 0.01 versus the Dv + H group.

The inhibition of YAP activity by VP and protein expression in lung tissues were examined by Western blotting. YAP expression was significantly lower in the VP group than in the Dv group (Figure 2A); moreover, the binding of YAP to β-catenin was also reduced in the VP group, as shown by immunoprecipitation (Figure 2B). After confirming the ability of VP to inhibit YAP activity, it was administered to mice, which were then subjected to HVT. The Dv + H group exhibited severe disruption of pulmonary architecture, extensive infiltration of inflammatory cells, alveolar hemorrhage and marked thickening of the alveolar septum, and these changes were further aggravated in the VP + H group (Figure 2C). HVT significantly reduced β-catenin expression in the cytoplasm; moreover, VP-mediated inhibition of YAP activity in combination with HVT, further decreased β-catenin levels (Figure 2D). Type II AECs can replace damaged type II AECs or type I AECs via proliferation or transdifferentiation; however, VP blocked this compensatory process. The expression of SP-C and AQP5 was decreased in the DV + H group compared with the Dv group, whereas the reduction in the expression of SP-C and AQP5 in the VP + H group was more significant than that in the Dv + H group (Figure 2E). And VP treatment aggravated lung injury and caused more severe pulmonary fibrosis. HVT caused diffuse perialveolar, peribronchial and interstitial fibrosis, which was aggravated in the mice treated with VP and subjected to HVT (Figure 2F).

**FIGURE 3:**
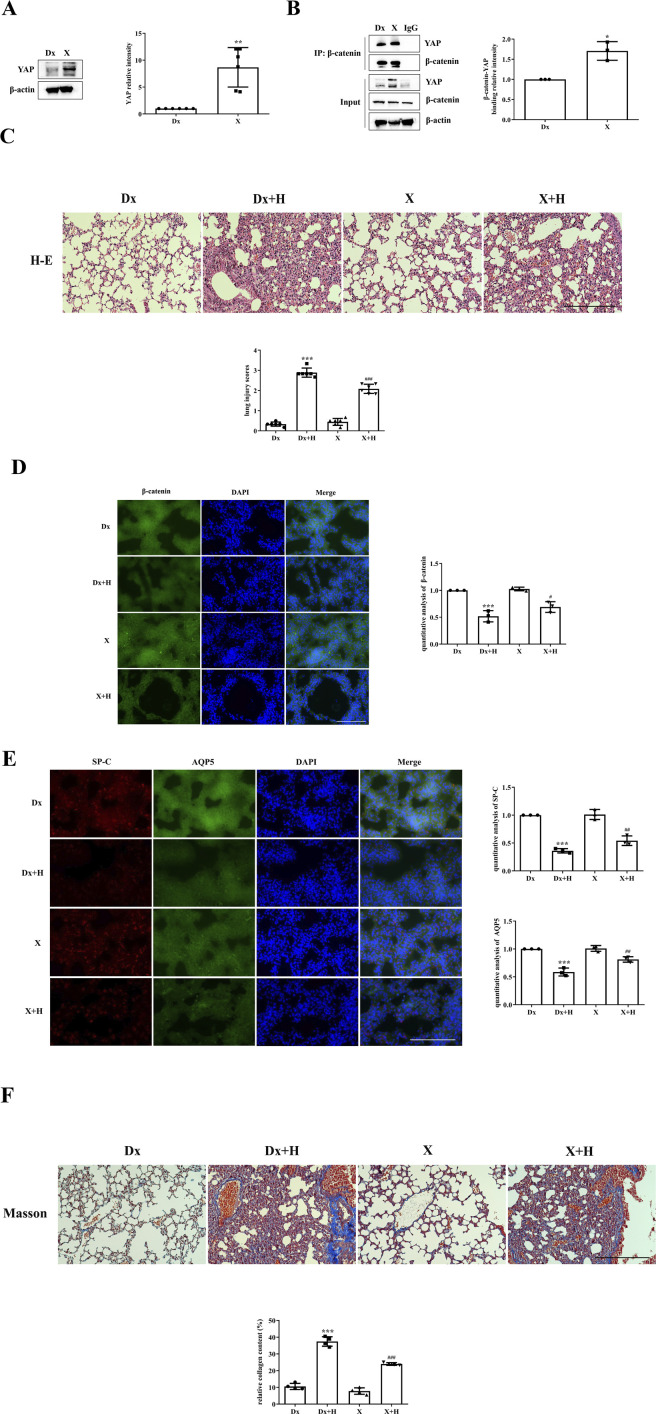
Stimulation of YAP activity alleviated VILI. **(A,B)** Mice were subjected to HVT and pretreated with or without XMU-MP-1, and YAP activity and the colocalization of YAP with β-catenin were measured by Western blotting (Dx group (1.000 ± 0.00), X group (8.672 ± 3.666)) and immunoprecipitation (Dx group (1.000 ± 0.00), X group (1.707 ± 0.2301)). Statistical analysis was performed. ^**^ *p* < 0.01, ^*^ *p* < 0.05 versus the Dx group. **(C)** H&E and lung injury scores (Dx group (0.3333 ± 0.1054), Dx + H group (2.889 ± 0.2277), X group (0.4444 ± 0.1721), X + H group (2.083 ± 0.2297)) were used to evaluate the severity of lung injury in mice subjected to HVT and pretreated with or without XMU-MP-1. Scale bar = 100 μm; magnification = ×200. ^***^ *p* < 0.001 versus the Dx group, ^###^ *p* < 0.001 versus the Dx + H group. **(D, E)** Frozen sections were fixed, permeabilized and incubated with anti-β-catenin or a mixture of anti-SP-C and anti-AQP5 primary antibodies followed by donkey anti-mouse (green) or donkey anti-rabbit (red) secondary antibodies, and the nuclei were stained blue. Scale bar represents 50 μm. The data are expressed as the means ± SDs, the expression of β-catenin in Dx group (1.000 ± 0.0), Dx + H group (0.5187 ± 0.1044), X group (1.026 ± 0.03480), X + H group (0.6901 ± 0.09927); the expression of SP-C in Dx group (1.000 ± 0.0), Dx + H group (0.3606 ± 0.03815), X group (1.012 ± 0.08959), X + H group (0.5424 ± 0.08624); the expression of AQP5 in Dx group (1.000 ± 0.0), Dx + H group (0.5865 ± 0.07133), X group (1.010 ± 0.05172), X + H group (0.8127 ± 0.04979). ^***^ *p* < 0.001 versus the Dx group, ^#^ *p* < 0.05, ^##^ *p* < 0.01 versus the Dx + H group. **(F)** Pulmonary fibrosis was assessed by Masson’s trichrome staining. Scale bar = 100 μm; magnification = ×200. Dx group (10.57 ± 1.842), Dx + H group (37.45 ± 2.836), X group (7.797 ± 1.920), X + H group (23.97 ± 0.8020). The relative collagen content was quantitatively analyzed, ^***^ *p* < 0.001 versus the Dx group, ^###^ *p* < 0.001 versus the Dx + H group.

YAP activity was stimulated by administering XMU-MP-1 to mice. Compared with that in the Dx group, the expression of YAP in the XMU-MP-1 treatment group was significantly greater (Figure 3A), and the colocalization of YAP and β-catenin was also greater (Figure 3B). Pathological changes associated with lung injury could be intuitively observed in the mice. While the Dx + H group presented severe disruption of pulmonary architecture, extensive infiltration of inflammatory cells, alveolar hemorrhage, thickening of the alveolar septum, and higher lung injury scores, these changes were mitigated in the X + H group (Figure 3C). The decrease in the localization of β-catenin was inhibited in the X + H group compared with the Dx + H group (Figure 3D); moreover, the reduction in the localization of SP-C and AQP5 was also inhibited (Figure 3E). Notably, the Dx + H group showed diffuse perialveolar, peribronchial and interstitial fibrosis, which was alleviated in X + H group (Figure 3F).

## Discussion

MV-induced activation of inflammatory signaling pathways mediates VILI (Ran et al., 2023), which is accompanied by the activation of lung repair processes and type II AECs proliferation or transdifferentiation into type I AECs to replace damaged AECs. If damage continues or worsens, incomplete lung repair, excessive deposition of extracellular matrix and the proliferation of fibroblasts can lead to pulmonary fibrosis. Inhibiting inflammatory responses and accelerating lung repair are expected to be effective therapeutic approaches for VILI.

The indications for HVT and LVT in clinical practice differ, as LVT has lung-protective effects, while HVT accelerates lung retention (Sousse et al., 2015; Nieman et al., 2020). The present study examined the effects of HVT and LVT on lung injury, YAP and β-catenin expression, lung repair and pulmonary fibrosis. VILI was found to be associated with an increase in YAP levels and a decrease in β-catenin levels; moreover, impaired lung repair and fibrosis occurred, and HVT had a stronger effect than did LVT. Ultimately, HVT was selected for subsequent experiments.

Wnt signaling pathways are classified as β-catenin-dependent classical pathways and nonclassical pathways and can regulate cell proliferation and differentiation during embryonic development (Liu et al., 2022; Wioletta et al., 2017). Other studies have shown that the hippo signaling pathway can regulate the Wnt/β-catenin signaling pathway and that changes in the expression and localization of YAP, the main transcriptional regulator of hippo signaling pathway components, can modulate cell proliferation and differentiation (Jia et al., 2021). Verteporfin is a YAP inhibitor that disrupts the YAP-TEAD interaction to induce apoptosis and is also an autophagy inhibitor that blocks autophagy at early stages by inhibiting autophagosome formation (Barrette et al., 2022). Suppression of YAP activity by Verteporfin exacerbated the decrease in the binding of YAP to β-catenin. After HVT, Verteporfin worsened lung injury, inhibited the compensatory regeneration of AECs, and further promoted the progression of pulmonary fibrosis.

The present study demonstrated that the suppression of YAP activity worsened the HVT-induced reduction in β-catenin expression, significantly reduced the compensatory regeneration and repair capacity of AECs, and aggravated lung injury and pulmonary fibrosis. CTGF is a cysteine-rich peptide secreted by various cells such as endothelial cells, fibroblasts, smooth muscle cells and myofibroblasts, and its expression is increased in various fibrotic diseases. The expression of CTGF, as the “master switch” of fibrosis, also increased after HVT. And HVT increased the expression of YAP, which attempted to compensate for VILI; however, Verteporfin interfered with this process, and the final outcome was a decrease in SP-C and AQP5 expression. Subsequently, lung injury persisted, lung repair was blocked, inducing pulmonary fibrosis. To further confirm the above conclusion, we stimulated YAP activity with XMU-MP-1, a selective Mst1/2 inhibitor that prevents YAP phosphorylation and stimulates its nuclear translocation and activity (Zhang et al., 2021). The expression of YAP and its binding with β-catenin increased after XMU-MP-1 treatment. Stimulation of YAP activity decreased the severity of lung injury caused by HVT, promoted the regeneration of AECs, and inhibited the development of pulmonary fibrosis. Thus, YAP activity can regulate lung injury, and stimulation of YAP activity alleviates VILI. This study has several limitations: the isolation of type I and II AECs during HVT is difficult, and the proliferation and transdifferentiation of AECs during this process cannot be dynamically observed; however, the protein levels of type II and I AEC markers can be measured instead.

Our research group reported in previous studies that HVT leads to VILI through biotrauma, which involves a cascade of inflammatory events. Early intervention in inflammatory signaling pathways, such as through the inhibition of NLRP3 inflammasome activation, can effectively inhibit VILI; moreover, promoting lung repair can also alleviate VILI. The mechanisms of lung repair are complex and involve many signaling pathways. In addition to the hippo and Wnt/β-catenin signaling pathways, the BMP/Smad and Notch signaling pathways are also involved in lung repair, and there may be mutual regulatory mechanisms among these signaling pathways (Zhao et al., 2013; Liu X. et al., 2020). Excessive lung repair leads to pulmonary fibrosis, that is, to refractory lung injury, which increases the mortality and hospitalization costs of VILI patients. Inhibiting the exacerbation of the inflammatory response in the early stage can accelerate lung repair, prevent pulmonary fibrosis, and effectively inhibit VILI. This study suggests that targeting YAP and stimulating YAP activity can promote the proliferation and transdifferentiation of AECs to alleviate VILI.

Our findings confirmed that MV caused VILI accompanied by increased YAP activity, inhibition of YAP activity accelerated VILI by suppressing AEC regeneration, and stimulation of YAP activity alleviated VILI and promoted the recovery of lung architecture. This study suggested that targeting YAP and stimulating YAP activity can promote the proliferation and transdifferentiation of AEC to alleviate VILI.

## Data Availability

The original contributions presented in the study are included in the article/supplementary material, further inquiries can be directed to the corresponding author.

## Abbreviations

AEC, Alveolar epithelial cell; CTGF, Connective tissue growth factor; HVT, High tidal volume ventilation; LVT, Low tidal volume ventilation; MV, Mechanical ventilation; TEAD, Transcriptional enhanced associate domain; VILI, Ventilator-induced lung injury; X, XMU-MP-1; YAP, Yes-associated protein; VP, Verteporfin.
